# Metabolizable Energy from Cashew Nuts is Less than that Predicted by Atwater Factors

**DOI:** 10.3390/nu11010033

**Published:** 2018-12-24

**Authors:** David J. Baer, Janet A. Novotny

**Affiliations:** USDA, ARS, Beltsville Human Nutrition Research Center, Beltsville, MD 20705, USA; janet.novotny@ars.usda.gov

**Keywords:** cashew, anacardium occidentale, digestibility, energy, calories

## Abstract

Recent studies have demonstrated that the energy provided by several tree nuts is less than that predicted by the Atwater factors, though energy available from cashews has never been assessed. The objective of this study was to evaluate the metabolizable energy in cashew nuts. Eighteen healthy adults were enrolled in a randomized, crossover study with two treatment periods. Subjects were fed a fully controlled base diet for 4 weeks with either no additions or with the addition of 42 g/day (1.5 servings) of cashew nuts, with the final treatment diets being isocaloric. Complete diet collections were analyzed for nitrogen (for protein), fat, energy, and carbohydrate by difference. During the final week of each intervention phase, subjects collected all feces and urine produced, and these were also analyzed for nitrogen (feces and urine), energy (feces and urine), and fat (feces). The resulting data were used to calculate the metabolizable energy of cashews and the digestibility of macronutrients. The average available energy (calorie) content of a 28 g serving of cashew nuts was 137 kcal (±3.4 kcal SEM) and ranged from 105 to 151 kcal. The mean value of 137 kcal/serving is 16% lower (*p* < 0.0001) than what is typically found on food labels. Digestibility of energy, fat, protein, and carbohydrate was lower for the cashew-containing diet compared to the control diet (92.9% vs. 94.9%, *p* < 0.0001 for energy; 96.1% vs. 97.8%, *p* = 0.0009 for fat; 90.1% vs. 91.2%, *p* = 0.0012 for protein; 92.9% vs. 94.9%, *p* < 0.0001 for carbohydrate; for the cashew-containing diet vs. the control diet, respectively). In conclusion, cashews provide fewer calories than the values predicted by the Atwater factors, as found on current food labels.

## 1. Introduction

For over 100 years, the Atwater factors have been used to calculate metabolizable energy (the calorie value that is typically found on food labels) in foods. However, recent evidence has demonstrated that the Atwater factors do not provide an accurate value for metabolizable energy in several tree nuts, including pistachios [1], almonds [2,3], and walnuts [4]. The inaccuracy of the energy value calculated using the Atwater factors is likely due to their overestimation of the digestibility of macronutrients from nuts. For nuts and legumes, the digestibility of protein, fat and carbohydrate are assumed to be 78%, 90% and 97%, respectively [5]. While the actual digestibility has not been reported in the literature, recent studies have found that the cellular structure of nuts limits digestion [6,7,8,9]. As a result of whole cells from nuts remaining intact in feces, and the fat vacuoles that they contain, fat is excreted and not digested [7]. Microscopic analyses and in vitro digestion studies suggest that the plant cell wall limits digestion of nuts. These observations suggest that the assumed digestibility of macronutrients is lower than the values used to calculate the metabolizable energy using the Atwater factors. Because the Atwater factors are the basis for the most common method for calculating available energy content of foods, this discrepancy has resulted in inaccurate food labels.

Accurate food labels are important for communicating nutrient and energy content of foods to consumers. The recent studies of metabolizable energy in pistachios [1], almonds [2,3], and walnuts [4] have provided a direct assessment of metabolizable energy for improved accuracy on food labels. Moreover, empirical data demonstrating that the energy density of nuts is less than previously thought serves to lower a barrier to consumption, which is the perception that tree nuts are high calorie foods. However, to date cashews have not been studied with respect to bioavailable energy.

Cashews (*Anacardium occidentale*), as with other tree nuts, are an important source of minerals, especially magnesium. Magnesium is a shortfall nutrient in the United States [10]. Similarly, in Europe, more than 70% of the Spanish population is not consuming adequate magnesium [11]. Also, cashews are an important commodity internationally and the fourth most consumed nut in the United States [12]. Consumption of tree nuts instead of other snack foods has been shown to improve nutrient intake [12]. If cashew nuts, as with other tree nuts, have lower energy digestibility and provide less energy than predicted, then this information may impact consumer choice in food selection.

A randomized crossover intervention was conducted with healthy adults with the primary objective of measuring the metabolizable energy value of cashews, and to compare the measured energy value with the energy value calculated using Atwater factors. A secondary objective of this intervention was to determine how adding cashews to the diet impacts the digestibility of dietary macronutrients. Subjects consumed a base diet with or without cashews, collecting all urine and feces produced for the last week of each intervention phase. Analysis of diets, urine, and feces were used to calculate the metabolizable energy content of cashew nuts and diet digestibility.

## 2. Materials and Methods

Volunteers were recruited to participate in a 2-phase, randomized crossover diet intervention study. For recruitment, an email advertisement was sent to names in the USDA Beltsville Human Nutrition Research Center (BHNRC) database and to Federal employees in the area near Beltsville, MD (near Washington, DC, USA). Interested volunteers provided written, informed consent, then completed a study application, health history questionnaire, and blood and urine screening, all of which were used to confirm eligibility. Volunteers were required to have a body mass index of between 20 and 38 kg/m^2^, be of age 25 to 75 years, have fasting glucose < 7.0 mmol/L, and have blood pressure ≤ 160/100 mm Hg. Volunteers were excluded from participation if they had gastrointestinal or metabolic diseases or disorders, presence of kidney disease, liver disease, gout, hyperthyroidism, untreated or unstable hypothyrioidism, certain cancers, pancreatic disease, diabetes, other metabolic diseases, use of cholesterol lowering medication, use of medication to treat hypertension for less than 6 months, active cardiovascular disease, pregnant or lactating or recently given birth (within past 12 months), use of antiobesity medications or supplements, recent or current smoker (within past 6 months) or substance abuser. The study protocol and all procedures were approved by Chesapeake Institutional Review Board, and all subjects provided written, informed consent. The study was conducted at the USDA Beltsville Human Nutrition Research Center in Beltsville, MD, USA. The trial was registered with www.clinicaltrials.gov (NCT02628171).

### 2.1. Study Design

The study was a sub-study of a larger study investigating the effect of cashew nuts on blood lipids [13]. For the primary study, 42 adults were enrolled in a randomized crossover trial with two 4-week feeding periods. The goal of the larger study was to evaluate the impact of consumption of 1.5 servings of cashew nuts daily on total cholesterol, LDL cholesterol, HDL cholesterol, and triglycerides, since cashew nuts have a different fatty acid profile from other nuts previously evaluated for blood lipid effects. Eighteen of those subjects were selected to participate in this sub-study of the metabolizable energy value of cashew nuts.

**Figure 1:**
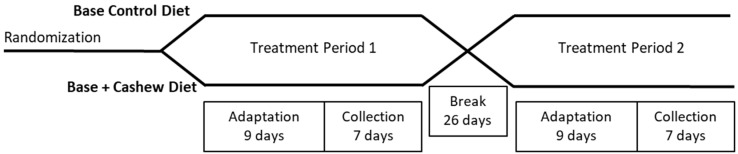
Study schematic indicating treatment periods, crossover design, adaptation of the diet and excreta collection periods.

For this randomized crossover trial, subjects consumed a fully controlled diet throughout the two 4-week feeding periods. During one period, the subjects consumed a base diet, and during the other period, subjects consumed the same base diet, adjusted to include cashew nuts (described below). During the last week of each feeding period, subjects collected all urine and feces, and delivered them to the USDA BHNRC (Figure 1). From the energy and macronutrients consumed and excreted, it was possible to calculate the metabolizable energy provided by the cashew nuts.

### 2.2. Diets

The base diet had a macronutrient profile of 33.3% en fat, 16.8% en protein, and 49.9% en carbohydrate. Upon enrollment, the energy requirement of each subject was calculated using a combination of the revised Harris-Benedict equation [14] and the Mifflin equation [15], multiplied times an activity factor determined from the activity description on the subject’s study application, and diets were scaled according to energy need. Subjects were weighed daily, and patterns of weight change were followed by adjustment in the prescribed diet to achieve weight maintenance. During the control phase (no cashew nuts), the base diet met each subject’s entire energy requirement. During the treatment phase, during which subjects consumed 42 g cashew nuts per day, all foods in the base diet were reduced proportionally such that the control base diet and the cashew-containing diet were calculated to be isocaloric. The amount of cashews consumed (42 g/day) is based on the amount specified by the US Food and Drug Administration’s qualified health claim for tree nuts. During the controlled feeding period, subjects were instructed to eat all the foods provided to them and only the foods provided to them.

Subjects consumed breakfast and dinner Monday through Friday at the USDA BHNRC Human Study Facility under the supervision of study staff. On weekdays, cashew nuts were provided with breakfast (21 g cashew nuts) and dinner (21 g cashew nuts) so that staff could observe consumption of the treatment food. Lunch and weekend meals were packed for carryout.

### 2.3. Sample Collection and Analysis

During the last week of each feeding period, duplicate trays of food were collected for analysis of macronutrients and energy. Each single day’s food was combined in a Waring blender, then freeze-dried and well mixed. Samples of composited diet were analyzed for fat, nitrogen, energy, and ash.

During the last week of the feeding period, each subject was instructed to collect all urine and feces produced. For urine collection, subjects were provided with a large collection vesicle and two 4-L pre-weighed jugs daily containing 10 g boric acid, as well as a cooler with dry ice. For fecal collection, subjects were given a supply of plastic zip-sealing bags and a toilet tray to hold the bags during defecation. A capsule containing the dye Brilliant Blue was given to each subject at the beginning of the collection period and after 7 days of collection to mark the beginning and end of each collection period. Feces were stored in the cooler with ice until delivery to the USDA BHNRC. A collection form was provided daily to record the date and time of each urine and fecal sample. Each morning, when reporting to the USDA BHNRC for breakfast, the subjects delivered their samples, then after breakfast they were given new collection supplies for the next 24-h period. Samples were analyzed for fat (feces), nitrogen (urine and feces), energy (urine and feces), and ash (feces). As previously reported, the macronutrient, sterol and fatty acid composition of the cashews were analyzed at a commercial laboratory (Covance Laboratory, Madison, WI, USA).

Gross energy was measured in diets, urine, and feces by adiabatic bomb calorimetry (Parr Instrument Company, Moline, IL, USA). Nitrogen in diets, urine, and feces was measured by the Dumas method (Leco Corp., St. Joseph, MI, USA). Fat content of diets and feces were measured by petroleum ether extraction (Soxtec, Foss, Eden Prairie, MN, USA). Ash was measured by combustion in a muffle furnace. Total carbohydrate in samples of diet and feces was calculated by difference from measured values for fat, protein, and ash in the dry matter. Analyses were performed in duplicate, and the mean of these values was used for statistical analyses.

### 2.4. Data Analyses

Sample size was determined based on data from previous studies conducted in our laboratory. A sample size of 18 subjects would be needed to detect a difference greater than 10% between the measured and calculated energy content of cashews. This sample size was calculated assuming a standard deviation of 0.87 for the difference between calculated and measured energy value (which is the average standard deviation that we have observed) with the criteria of 80% power and a *p*-value of 0.05.

Calculation of metabolizable energy from cashews was performed as previously described [1]. Excreta composition and nutrient apparent digestibility ((intake − excreta)/intake × 100) were analyzed using a mixed model (PROC MIXED, SAS version 9.4, Cary, NC, USA) with subject (sequence within subject) as the random effect. Age, BMI, sex, treatment period, and treatment sequence were included in the model as stratification covariates. Interaction terms of these covariates with treatment were also included and removed from the model if not significant (no interactions were significant). Different covariance structures were evaluated, and the model fit was evaluated using the Bayesian information criteria. The variance components covariance structure was selected. Data were evaluated for normality using PROC UNIVARIATE. Changes in mean body weight between the beginning and end of the two treatments periods were evaluated using PROC TTEST.

## 3. Results

### 3.1. Results from Recruitment and Enrollment of Study Subjects

**Figure 2:**
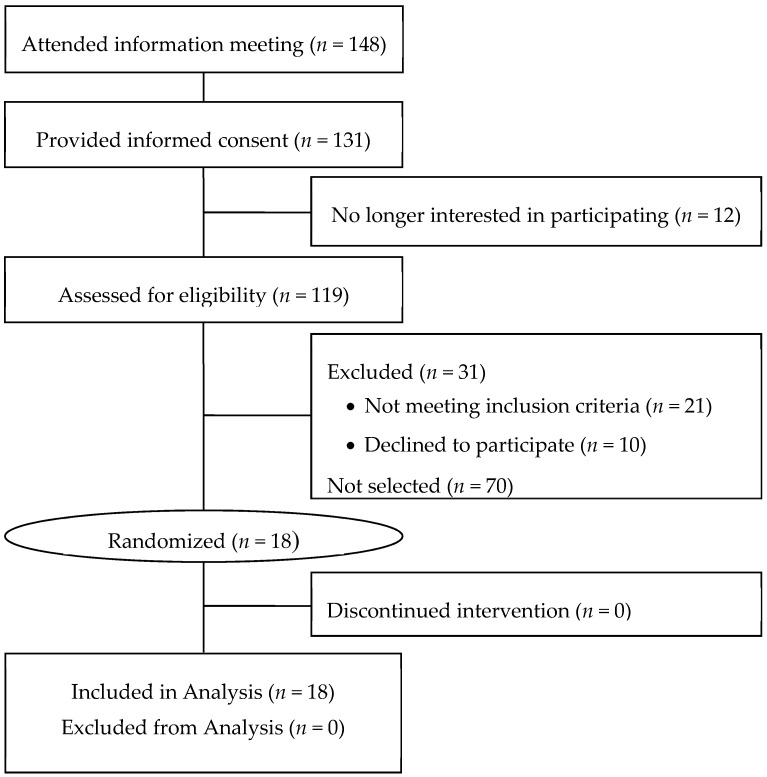
CONSORT (Consolidated Standards of Reporting Trials) diagram for the study of metabolizable energy provided by cashew nuts.

Eighteen subjects were enrolled in this this study. All subjects who were randomized completed the study, and data were included from all subjects in the analyses (Figure 2). Mean (± standard error) age of the 9 men and 9 women was 56.9 ± 2.4 years, and their BMI was 28.4 ± 1.1 kg/m^2^. Other characteristics just prior to the start of the intervention (“baseline”) are presented in Table 1.

### 3.2. Composition of Cashew Nuts

Macronutrient, fatty acid, and sterol content of the cashew is presented in Table 2 (adapted from [13]). Fat content was 29.8% of energy, carbohydrate content was 21.5% of energy and protein content was 11.4% of energy. Monounsaturated fatty acids were 16.85% of energy with oleic acid being the most abundant fatty acid. Saturated fatty acids were 5.98% of energy and polyunsaturated fatty acids were 5.32% of energy. Total sterols were 77.5 mg/100 g and beta-sitosterol was the most abundant sterol.

### 3.3. Metabolizable Energy of Cashew Nuts

**Figure 3:**
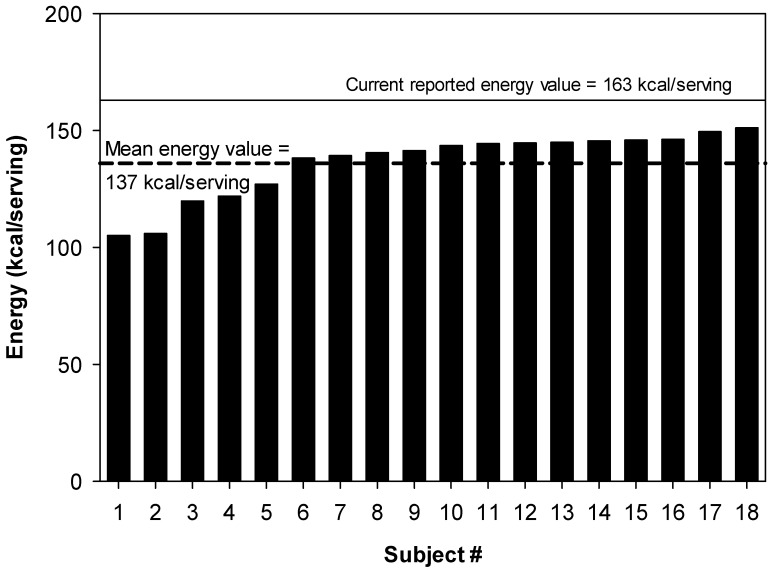
Energy value of cashew nuts. The dash line indicates the measured mean metabolizable energy value of 128.4 g serving of cashews. The solid line indicates the current value of cashews calculated using Atwater factors [16]. Subject # indicates data from an individual subject; each subject number indicates a different individual.

The available energy content of cashews was calculated for each research volunteer. The mean metabolizable energy value of a 28 g serving, and the energy value from each individual are presented in Figure 3. The average metabolizable energy (calorie) content of a 28 g serving was 137 kcal (±3.4 kcal SEM) and ranged from 105 to 151 kcal (Figure 3). The mean value of 137 kcal/serving was 16% lower (*p* < 0.0001) than what is typically found on food labels in the United States as well as the value (163 kcal/serving) that is found in USDA National Nutrient Database for Standard Reference [16] (NDB #12085, Nuts, cashew nuts, dry roasted, without salt added). In kJ, the mean value was measured to be 573 kJ with a range from 439 to 632 kJ, compared to a U.S. food label value of 670 kJ. Furthermore, the highest metabolizable energy value for an individual subject (151 kcal/28 g serving) was less than the current label value (Figure 3).

### 3.4. Body Weight

Body weight of the subjects did not change between the beginning and end of the two treatment periods. Mean body weight (± SE) at the beginning of the Base Control Diet treatment was 83.1 ± 3.8 kg (Table 1) and mean body weight at the end of that treatment was 82.8 ± 3.7 kg (*p* = 0.15). Mean body weight (± SE) at the beginning of the Base+Cashew Diet treatment was 83.1 ± 3.8 kg and mean body weight at the end of that treatment was 83.0 ± 3.8 kg (*p* = 0.67). These results confirm that subjects were at weight maintenance over the intervention period.

### 3.5. Macronutrient Intake and Absorption

Addition of cashews to the base diet altered the macronutrient distribution (Table 3). Intake of the cashew-containing diet provided 13 grams more fat on average, and 10 grams less carbohydrate on average. Protein intake between the two treatments was not affected by the addition of cashews. There was a small but statistically significant difference in gross energy intake between the two treatment diets (80 kcal/day). This difference is in part due to adjustments made to food intake to achieve weight stability during the two treatment periods. This small difference in gross energy intake between the two treatment periods did not affect body weight.

The macronutrient and energy content of the excreta changed with addition of cashews to the diet (Table 4). With the addition of cashews to the diet, fecal dry weight increased (*p* < 0.0001) from 27.0 to 34.2 g/day (±1.5 g/day), fecal fat increased (*p* = 0.0003) from 1.7 to 3.6 g/day (±0.3), fecal nitrogen increased (*p* = 0.0051) from 1.5 to 1.7 g/day (±0.1), and fecal carbohydrate increased (*p* < 0.0001) from 10.7 to 14.2 g/day (±0.6). Concomitant with the increase with fecal fat, protein and carbohydrate, fecal energy increased from 129.6 kcal/day to 186.3 kcal/day (±8.1) with the addition of cashews to the diet. Daily urinary energy (118.2 and 115.9 kcal/day, after consumption of the Base Control Diet and the Base + Cashew diet, respectively) and daily urinary nitrogen excretion (13.5 and 13.6 g/day after consumption of the Base Control Diet and the Base + Cashew diet, respectively) were not different between the two diets.

Diet (total diet, not cashew) digestibility was altered by the addition of cashews to the diet (Table 5). When cashews were consumed, the digestibility of the diet decreased for energy, fat, protein, and carbohydrate (Table 5). With the addition of cashews to the diet, percent fat digestibility decreased (*p* = 0.0009) from 97.8% to 96.1% (±0.3), percent protein digestibility decreased (*p* = 0.0012) from 91.2% to 90.1% (±0.5), and percent carbohydrate digestibility decreased (*p* < 0.0001) from 94.9% to 92.9% (±0.2). Concomitant with the decrease in dietary fat, protein, and carbohydrate digestibility, energy digestibility of the diet also decreased (*p* < 0.0001) with the addition of cashews to the diet from 94.9% to 92.9% (±0.2).

## 4. Discussion

The primary outcome for this study was the metabolizable energy (energy available to the body, accounting for fecal and urinary losses) of cashew nuts. The metabolizable energy value of a serving of cashews is 16% lower than predicted using Atwater factors. This observation is consistent with data on other tree nuts—that the calorie value on food labels overestimates that actual available calories.

The overall diets with and without cashews were designed to be isocaloric based on calculations of energy content of the foods provided. Body weight was monitored daily to confirm that subjects remained at weight maintenance. For four subjects, changes in body weight were observed during the first weeks of the study, leading to small adjustments in their energy intake to achieve weight stability. As a result, mean total gross energy intake for the study cohort was slightly greater during the Base+Cashew Diet period than during the Base Control Diet period. Despite this small difference in gross energy intake, body weight was not different at the end of the two treatment periods. Moreover, the method for determining the metabolizable energy of cashews accounts for the differences in gross energy intake and fecal and urinary output between the two treatment periods.

Previous studies in our laboratory demonstrated that for almonds and walnuts, the difference between predicted and measured metabolizable (available) energy is 20 to 25% [2,3,4], whereas for pistachios [1], the difference is about 5%. Furthermore, we have shown that the extent of processing of almonds affects their metabolizable energy content. Roasting and homogenizing almonds increases their metabolizable energy value [2]. Roasting increases the brittleness of the nut, and mastication of these more brittle nuts results in smaller particles [2]. These smaller particles have an increased surface area-to-volume ratio, and exposes the cell wall contents to digestive enzymes. Homogenizing (i.e., almond butter) also results in smaller particles and increased nutrient digestibility [2]. The phenomenon occurring in these cases is that the cell wall, which is resistant to digestion, is being disrupted. Through this disruption of the cell wall, cellular contents, including fat vacuoles, are made available for small intestine enzymatic digestion.

The digestibility of the diet decreased when cashews were consumed as part of the menu. This reduction in diet digestibility by the addition of tree nuts was observed previously for almonds [2,3], walnuts [4], and pistachios [1]. A previous study with almonds demonstrated that more lipid remained encapsulated within plant tissue for whole or diced almonds compared to almond butter, suggesting that digestion of almonds is limited by the plant structure [8]. Lipid bioaccessibility is indirectly associated with tree nut particle size, as demonstrated by an in vitro model of duodenal lipolysis of almonds [17]. It is likely that the same phenomenon occurs for other tree nuts, including cashews.

The impression of a calorically dense food can be an impediment to its consumption [18,19]. In contrast, the demonstration that cashews provide less metabolizable energy than previously thought could remove a barrier to cashew consumption, opening the possibility of reaping health benefits from their consumption. One such health benefit is the provision of magnesium. Magnesium is a shortfall nutrient in the U.S. [10], with approximately 50% of adults not meeting the Estimated Average Requirement (EAR) for magnesium [20], and more than 70% of individuals in Spain not consuming adequate amounts of magnesium [11]. Daily intake of a single serving of cashews per day would reduce the proportion of U.S. adults in the inadequate range from 56% to less than 25% for women and from 53% to less than 25% for men [16,20]. Consumption of 1.5 servings of cashew nuts per day would reduce the percent of adults not getting enough magnesium to less than 5% for women and less than 10% for men [16,20].

The strengths of this study are the highly controlled feeding using a paired-diet approach, adaptation to the diet prior to collecting the fecal and urine samples, and the length of the collection period. These factors minimize errors and improve the measurement of the metabolizable energy content. A limitation of this study is that only one dose of cashews was fed limiting insights into dose-effects. However, in previous research with almonds [3], there was no difference in the metabolizable energy value of almonds when consumed daily as 42 g or 84 g. Additional research with cashews is needed to understand dose-effects.

## 5. Conclusions

In conclusion, cashew nuts provide fewer calories than that predicted by the Atwater factors and the value used on food labels. In addition, a diet containing cashew nuts has lower digestibility than the same diet without cashew nuts. This finding is in accord with published results for other tree nuts. The understanding that cashews and other tree nuts provide fewer calories than previously thought may help to remove an obstacle to the consumption of these nutrient dense foods.

## Figures and Tables

**Table 1:** Characteristics of study subjects prior to the intervention (*n* = 18, 9 men and 9 women).

Characteristic	Mean ± SE
Age (year)	56.9 ± 2.4
Body weight (kg)	83.1 ± 3.8
BMI (kg/m^2^)	28.4 ± 1.1
Waist circumference (cm)	100.9 ± 2.7
Total cholesterol, fasting (mg/dL)	205.2 ± 5.8
Triglycerides, fasting (mg/dL)	114.6 ± 14.0
Glucose, fasting (mg/dL)	96.3 ± 2.0
Systolic blood pressure (mm Hg)	119.6 ± 3.9
Diastolic blood pressure (mm Hg)	72.6 ± 2.1

BMI: Body Mass Index; SE: Standard Error.

**Table 2:** Analyzed macronutrient, fatty acids, and sterol composition of the cashew nuts. Adapted from [13].

Nutrient	Mean ± SE
Macronutrients	
Fat (en%)	29.8 ± 0.01
Carbohydrate (en%)	21.5 ± 0.02
Protein (en%)	11.4 ± 0.04
Fatty acids	
Saturated fatty acids	5.98 ± 0.01
Palmitic acid (16:0) (en%)	2.94 ± 0.01
Stearic acid (18:0) (en%)	2.72 ± 0.00
Other saturated fatty acids (en%)	0.32 ± 0.00
Monounsaturated fatty acids	16.85 ± 0.08
Oleic acid (18:1) (en%)	16.59 ± 0.08
Other monounsaturated fatty acids (en%)	0.26 ± 0.00
Polyunsaturated fatty acids	5.32 ± 0.11
Linoleic acid (18:2) (en%)	5.28 ± 0.11
Other polyunsaturated fatty acids (en%)	0.05 ± 0.00
Total Sterols (mg/100 g)	77.5 ± 5.1
Campesterol (mg/100 g)	5.0 ± 0.0
Beta-Sitosterol (mg/100 g)	63.8 ± 5.1
Other sterols and stanols (mg/100 g)	8.8 ± 0.0

en%: Percent of energy.

**Table 3:** Gross energy and macronutrient intake of diets for the Base Control Diet and the Base + Cashew Diet.

	Base Control Diet (Mean, *n* = 18)	Base + Cashew Diet (Mean, *n* = 18)	Pooled SEM	*p* ^1^
Gross energy (kcal/day)	2530	2610	78	0.0172
Fat (g/day)	77.7	90.9	2.4	<0.0001
Nitrogen (g/day)	16.9	17.0	0.5	NS
Protein (g/day)	105.8	104.9	3.3	NS
Carbohydrate (g/day)	316.5	305.6	9.8	0.0104

^1^*p*-values < 0.05 were considered to be statistically significant. NS indicates non-significance.

**Table 4:** Composition of excreta for the Base Control Diet and the Base + Cashew Diet.

	Base Control Diet (Mean, *n* = 18)	Base + Cashew Diet (Mean, *n* = 18)	Pooled SEM	*p* ^1^
Fecal weight (g dry weight/day)	27.0	34.2	1.5	<0.0001
Fecal gross energy (kcal/day)	129.6	186.3	8.1	<0.0001
Fecal fat (g/day)	1.7	3.6	0.3	0.0003
Fecal nitrogen (g/day)	1.5	1.7	0.1	0.0051
Fecal protein ^2^ (g/day)	9.4	10.5	0.6	0.0051
Fecal carbohydrate ^3^ (g/day)	10.7	14.2	0.6	<0.0001
Urinary energy (kcal/day)	118.2	115.9	5.0	NS
Urinary nitrogen (g/day)	13.5	13.6	0.4	NS

^1^*p*-values < 0.05 were considered to be statistically significant. ^2^ Fecal protein calculated from fecal nitrogen as N × 6.25. ^3^ Fecal carbohydrate calculated by difference from fecal protein and fecal fat.

**Table 5:** Digestibility of the Base Control Diet and the Base + Cashew Diet.

	Base Control Diet (Mean, *n* = 18)	Base + Cashew Diet (Mean, *n* = 18)	Pooled SEM	*p* ^1^
Energy (%)	94.9	92.9	0.2	<0.0001
Fat (%)	97.8	96.1	0.3	0.0009
Protein ^2^ (%)	91.2	90.1	0.5	0.0012
Carbohydrate (%)	94.9	92.9	0.2	<0.0001

^1^*p*-values < 0.05 were considered to be statistically significant. NS indicates non-significance. ^2^ Protein digestibility calculated from nitrogen balance.
